# Experiences With Integrating Medical Terminologies Into User Interfaces for a Decision Support System for Primary Care: Conceptual and Development Study

**DOI:** 10.2196/74934

**Published:** 2026-02-20

**Authors:** Michaela Christina Neff, Jannik Schaaf, Najia Ahmadi, Michele Zoch, Dania Schütze, Holger Storf

**Affiliations:** 1 Institute of Medical Informatics University Medicine Goethe University Frankfurt Frankfurt Germany; 2 Institute for Medical Informatics and Biometry Carl Gustav Carus Faculty of Medicine Technical University of Dresden Dresden Germany; 3 Institute of General Practice Goethe University Frankfurt Frankfurt Germany

**Keywords:** user computer interfaces, primary care, clinical decision support, controlled vocabulary

## Abstract

**Background:**

Clinical decision support systems (CDSSs) have shown promise in improving diagnosis in primary care, particularly for chronic diseases. The SATURN (Smart Physician Portal for Patients With Unclear Disease) project developed a CDSS prototype for primary care in Germany that uses artificial intelligence to reduce diagnostic uncertainty in unclear and rare diseases. It generates recommendations based on clinical data from university hospitals stored in a standardized common data model. However, integrating primary care data in Germany remains challenging due to the use of country-specific vocabularies and heterogeneous data structures. Therefore, integration of medical concepts into general practitioners’ user interfaces (UIs) and improved workflow design is needed.

**Objective:**

This study investigates how the UI of a CDSS for primary care should be designed to facilitate the user-friendly entry of medical concepts.

**Methods:**

A structured, iterative user-centered design process of five steps was applied: (1) conceptualization, including analysis of requirements and objectives, (2) implementation of the CDSS UI, (3) analysis of user and expert feedback, (4) workshop with experts in the primary care electronic health record system, and (5) development of an extended concept for the system UI.

**Results:**

This study identified requirements and options for supporting data entry in the UI of a CDSS for primary care, thus providing a framework for future implementation and prototype development. Usability testing revealed the need to refine input support, particularly the language used to make the system easy for general practitioners to use. Further effort is required to map this. In addition, we have identified a system interface to electronic patient records in primary care as essential for the system.

**Conclusions:**

This paper demonstrates how standardized medical terminologies can be integrated into a CDSS UI for primary care. The user-centered design approach has been effective in developing CDSS UIs that align with clinicians’ workflows and provide a user-friendly experience. Although the current technical infrastructure presents significant challenges when connecting to primary care electronic health record systems, collaborating with experts enabled us to identify potential interface solutions. Overall, the results indicate a need for optimized physician language input support and interoperability, including standardized data entry, automated tools, and artificial intelligence–based approaches to enhance data quality and usability.

## Introduction

### Background and Significance

The use of clinical decision support systems (CDSSs) has been demonstrated to enhance diagnostic accuracy in primary care settings [1,2]. However, the prevailing focus at present is on chronic diseases [1]. In primary care, general practitioners (GPs) play a unique role in the diagnostic and referral processes. They often deal with nonspecific symptoms that may indicate a wide range of potential diagnoses, leading to diagnostic uncertainty. It is therefore essential to provide GPs with adequate support to ensure patient care [3-5]. Although the potential of CDSSs to achieve significant improvements in outcomes has been demonstrated, wide variations remain in the types and methods of CDSS implementation as well as the resulting effectiveness. Further research is required to determine effective implementation strategies for the usage of CDSS in various settings [6].

The SATURN (Smart Physician Portal for Patients With Unclear Disease) project has developed a prototype of a CDSS to address unclear diagnoses in primary care in Germany [7,8]. The development was conducted through an iterative, user-centered development process (UCD) [8]. The CDSS generates recommendations for GPs using 3 artificial intelligence (AI) modules based on clinical data (a machine learning algorithm and a case-based reasoning algorithm) and expert knowledge (a rule-based system). Clinical databases (rather than primary care databases) were used to establish connections between GPs and the knowledge data held by the university medicine. The diagnostic recommendations derived from clinical data are based on comparisons with existing cases [7,8]. Specifically, the data for these existing cases were extracted from the electronic health record (EHR) data of the University Hospitals in Frankfurt and Dresden via Data Integration Centers (DICs). The DIC is a central institution responsible for making medical data from hospital patient care accessible for medical research and clinical exchange in a structured, standardized, and pseudonymized form [9]. The CDSS database has been designed in such a way that EHR data from the 2 hospitals are merged into 1 OMOP database and can be used for the AI modules. Only data, not AI logic, is stored in the database. The AI modules are designed as separate modules.

The OMOP Common Data Model (CDM) is an open-access standardized data model [10] that can be used to ensure interoperability. The OMOP CDM harmonizes medical databases through a uniform structure and standardized terminologies such as SNOMED CT (Systematized Nomenclature of Medicine Clinical Terms) and RxNorm (normalized names for clinical drugs) [10-12]. New EHR patient cases from primary care for which a diagnosis recommendation is to be made will also be stored in this database, along with corresponding flags (flag: without diagnosis). In addition, solved patient cases are stored (flag: with final diagnosis). As the CDSS uses OMOP and is used in primary care, the data entered by primary care GPs must be compatible with the OMOP vocabulary standards. However, the use of standardized vocabulary remains a challenge, particularly in Germany’s primary care sector, where the predominant classification of diseases is *ICD-10-GM* (*International Statistical Classification of Diseases and Related Health Problems, Tenth Revision, German Modification*). *ICD-10-GM* is complemented by OPS (Operation and Procedure Codes; German: “Operationen- und Prozedurenschlüssel”) for procedural coding procedures [13], along with “Einheitlicher Bewertungsmaßstab” as the basis for billing GPs and contract psychotherapists [14]. Heterogeneous data structures in different countries and country-specific vocabularies (such as *ICD-10-GM*) pose significant challenges for exchanging data and comparing results. To our knowledge, there has been no full systematic integration of primary care data into the OMOP CDM, to date, in Germany. The standardization of data could facilitate the development of common tools. Initial international approaches have shown potential but are limited in their scope [15,16]. Henke et al [17,18] investigated the merging and collaboration of primary care and clinical data in Germany. These studies linked data from the DICs of the Medical Informatics Initiative with routine health insurance data and aimed to increase the coverage of the German vocabulary in the OMOP CDM. Another study examined the cross-sector linkage of primary care data and identified challenges, including system interfaces and data extraction processes [19]. Such projects show the need for using CDMs, such as the OMOP CDM and standardized vocabularies, to harmonize cross-sector data. To integrate medical concepts into GP user interfaces (UIs), the UI must be designed to support and streamline existing workflows [20]. Medical concepts are coded according to standards for terminology, listed in terminology management or the OMOP vocabularies.

### Objectives

The objective of this study is to explore how the UI of a CDSS for primary care should be designed to allow the entry of medical concepts in a user-friendly way. To our knowledge, it has not yet been investigated how a CDSS UI for primary care can be comprehensively mapped to enable the standardized data collection and integration of data in Germany. This study outlines the requirements and the available options for CDSS UI for primary care, providing both a technical overview and a conceptual framework to support future CDSS implementations. The CDSS UI implementation focuses on medical vocabularies and the input into the CDSS. Other components of the CDSS, and the iterative conception and development, are covered in previous publications [8,21,22].

## Methods

### Study Design

**Figure 1:**
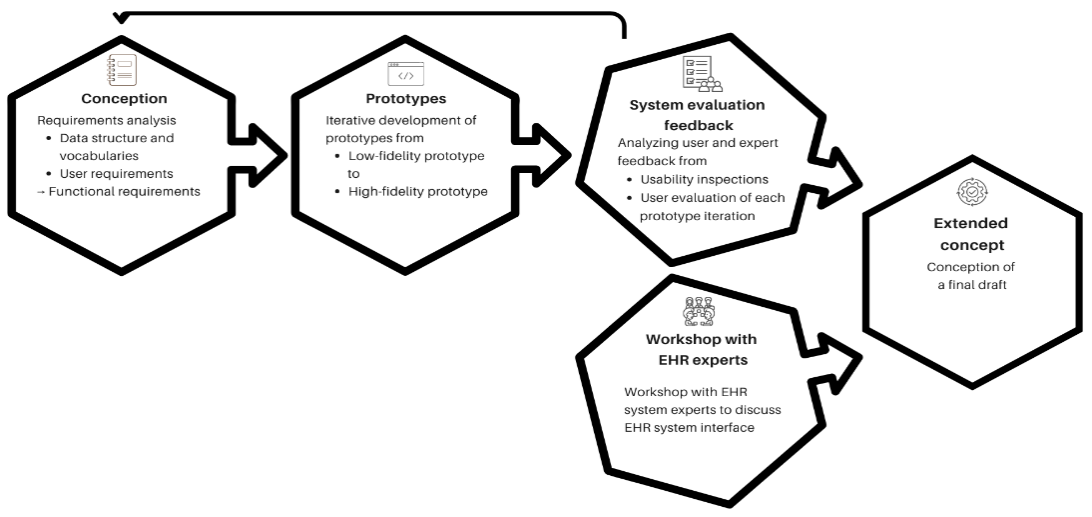
Methodological steps of this study for the development process of the CDSS data entry UI. CDSS: clinical decision support system; EHR: electronic health record; UI: user interface.

As in the whole SATURN project, an iterative UCD was used [23]. In this paper, we focus on data entry into the CDSS (Figure 1). The requirement analysis and underlying data model [24], the ongoing evaluation, as well as other parts of the CDSS, are described in previous publications [8,21,22,25]. The UCD, from the perspective of data input, began with a conceptual design stage. During this stage, the GPs’ requirements and necessary medical concepts were analyzed. These concepts were then coded into vocabularies and mapped to the UI design (step: conception). The UI then underwent iterative development in dialogue with the GPs (step: prototypes), followed by the collection of feedback from users and experts to evaluate its usability and to identify potential improvements (step: system evaluation feedback). EHR experts discussed the automation of medical concept entry (step: workshop with EHR experts). All participant groups were selected using a purposeful sampling approach. Finally (step: extended concept), a draft for the final design concept was created.

### Ethical Considerations

For the conception and development of this study, the SATURN project was previously approved by the Ethics Committee of Goethe University’s Medical Faculty (case 2022-1088). The user evaluation was also approved, with the Ethics Committee waiving the need for a separate ethics vote on the inquiry of GPs (case 2022-629). All participants received written information on the study and provided a signed informed consent. All data were pseudonymized before analysis. Participants received €150 (average currency exchange rate in August 2023: US $1=€0.92) for taking part in the usability test.

Participants in the usability inspections and the EHR expert workshop were either project participants or had previously submitted a letter of intent to support the project. All data were pseudonymized before analysis, with only sample data being used for case studies. Participants received no compensation for their participation in the usability inspections.

### Conception

In order to facilitate data entry into the CDSS, it is necessary to consider the structure and usage of the vocabularies, as well as the requirements of the end users [21]. The appropriate fields and variables must be defined with the vocabularies for the UI. The data model was defined in a previous study [24] and specifies the following vocabularies, shown in Table 1 [26-37].

All vocabularies shown in Table 1 are fully available in the German language, except for SNOMED CT and Human Phenotype Ontology (HPO). The data to be captured from the requirements and the data model, including its vocabularies, needed to be mapped to the UI and the fields of the UI. To accomplish this, UI data categories were created: basic patient data (patient ID, gender, date of birth, and postcode), diagnosis, symptoms, medication, vital and laboratory values, and procedures. The representation in the UI was described as a UI component with an input format. Where possible, mapping to an OMOP standard concept, such as SNOMED CT and RxNorm, was also ensured and documented. Data in the OMOP CDM are expressed as concepts, which represent the semantic meanings of each data element. A standard concept represents a normative expression of a clinical entity. The others were labelled as nonstandard or source concepts and mapped to the standard concepts [38]. In order to consider the user requirements and data input structure, these were translated into functional requirements. The user requirements collected [21] were analyzed for this purpose and transformed into functional requirements with specific, testable functions that the system should fulfil. Subsequently, use cases and functional requirements were formulated in the following template: [actor] must/should/will [conditions, time aspect] [object] [process description] or something more general [target system] + [priority] + [functionality] + [conditions] [39]. Thus, an example of a functional requirement could be “the system must enable the data to be exported in a usable format.” MCN, a medical informatician, carried out the conceptualization step in consultation with the other authors.

**Table 1:** Selected vocabularies according to the data model for the CDSS^a^.

Vocabulary	Description	Availability of the German translation	Progress or version of the German translation in August 2024
*ICD-10-GM* ^b^	Official classification for the encoding of diagnoses in inpatient and outpatient medical care in Germany [26]	Available	*ICD-10-GM* version 2024
HPO^c^	Standardized vocabulary of phenotypic abnormalities encountered in human disease [27]	Translation is not fully available in the German language	The HPO browser allows the display of some German HPO terms or synonyms [28,29], and a study on the translation of HPO terms was conducted as part of the project [30]
LOINC^d^	Database and universal standard for identifying medical laboratory observations [31]	Available	LOINC version 2.76
ATC/DDD^e^	Official classification for pharmacologically active substances. Active substances are divided into different groups according to the organ or organ system on which they act and according to their anatomical, therapeutic, and chemical properties. [32,33]	Available	ATC/DDD version 2024
OPS^f^	Official German classification for the encoding of operations, procedures, and general medical measures [34]	Available	OPS version 2024
SNOMED CT^g^	Systematized Nomenclature of Medicine Clinical Terms [35]	Translation is not fully available in the German language	The German translation group has been actively working on this since 2020 and translated the first national edition with SNOMED CT content into German at the end of 2023 [35-37]

^a^CDSS: clinical decision support system.

^b^ICD-10-GM: International Statistical Classification of Diseases and Related Health Problems, Tenth Revision, German Modification.

^c^HPO: Human Phenotype Ontology.

^d^LOINC: Logical Observation Identifiers Names and Codes Names and Codes.

^e^ATC/DDD: Anatomical Therapeutic Chemical Classification With Defined Daily Doses.

^f^OPS: Operation and Procedure Codes (German: “Operationen- und Prozedurenschlüssel”).

^g^SNOMED CT: Systematized Nomenclature of Medicine Clinical Terms.

### Prototypes

The functional requirements defined in the concept were developed and implemented by MCN iteratively (described in more detail in Neff et al [8]) while feedback from users and experts was collected through user evaluations [40,41] and usability inspections [8] (described in section “system evaluation feedback”).

The following intermediate (results) were created in the iterations: (1) low-fidelity prototype (CDSS version 0) [22], (2) first high-fidelity prototype (CDSS version 1) [8], and (3) final high-fidelity prototype (CDSS version 2).

The UI and the vocabulary used were implemented in German wherever possible to suit the German-speaking target. SNOMED CT was used in English due to its limited availability, and HPO was used in an initial partial translation by Noll et al [30]. To ensure good usability, design principles and usability guidelines [42-44] were followed, in addition to the points mentioned above, including principles of dialogue design in accordance with ISO (International Organization for Standardization) 9241 and other ISO standards [43,45,46].

### System Evaluation Feedback

The usability of the data entry process was assessed after each developed prototype. Initially, CDSS version 0 was discussed in 2 online user evaluation workshops involving 5 GPs, using a videoconferencing tool. This workshop was conducted and moderated by MCN with support from researchers from the Institute of General Practice (Frankfurt), and key statements were derived [22]. The findings were then documented as user requirements, and new functional requirements were derived where necessary. Usability expert inspections (method: heuristic walkthrough) were carried out in-person by 5 usability experts from the project team to accompany the further development of CDSS versions 1 and 2 (4 inspections conducted and analyzed by MCN: CDSS version 1: 3, CDSS version 2: 1) [8]. Finally, 2 usability evaluations were conducted for CDSS version 1 and version 2. Five GPs who participated in the version 0 evaluation took part in the version 1 evaluation, and 10 GPs (5 of whom were newly recruited) took part in the version 2 evaluation. The evaluations used the thinking-aloud test method, a postsession interview, and the system usability scale. The tests were conducted online by researchers from the Institute of General Practice (Frankfurt) with support from MCN [40].

The tasks that the users were asked to perform in the user evaluation and expert inspections were based on the task model derived from the requirements analysis: perform data entry, review and discuss AI results, plan further diagnosis, refer to specialists, and close the case [21]. The feedback from the expert inspections was categorized as bugs, features, improvements, ideas, or usability heuristics and linked to the individual steps of the task model [8]. When analyzing the feedback from the user evaluations, the usability problems identified in the usability tests were categorized as layout, content, navigation, comprehensibility, and usability, and also assigned to the steps of the task model [40]. This study focuses on analyzing and interpreting the evaluation results, particularly those concerning the task of “performing data entry.” The results of this task were analyzed to inform further development. The analysis of feedback represents an important conceptual intermediate step. Moreover, together with considerations regarding the connection to the primary care EHR system (see Workshop With EHR System Experts), it forms the basis for the final data entry concept within the CDSS.

### Workshop With EHR System Experts

As the interface to the primary care EHR system was identified as a key requirement in the analysis and subsequent evaluation iterations, a research workshop was conducted. This workshop was held in a hybrid format, combining in-person participation with remote connections via videoconference [47]. Research workshops focus on analyzing specific cases and collecting reliable and valid data on a participant’s future, including organizational change and design. These workshops yield theoretical concepts, methods, and applications that contribute to the discipline. The method involves presenting topics, conducting experiments, and then discussing the findings [48]. The central theme of the workshop was as follows: What should an interface between an EHR system and a decision support system for primary care look like? What is the current state of the art, and what is the vision for the future? The workshop focused on the technical possibilities and challenges of an interface between the CDSS and the EHR system in primary care. The participants were experts in this field, and no GPs were included. However, these experts served as representatives, as it is very difficult to cover the entire spectrum of the system landscape in Germany.

In more detail, the workshop was conducted with (1) project representatives of the SATURN project; (2) representatives from three different companies in the field of EHR systems for primary care, who are considered experts in EHR systems in this study; and (3) two moderators (with medical and medical informatics backgrounds) and one SATURN team member who took notes.

The discussion was supported by a structured discussion guide (Multimedia Appendix 1). This guide addressed the organizational, syntactic, semantic, and structural interface levels of a software. Furthermore, the UI data categories for the decision support system (see description of data categories in the chapter “conception”) with related vocabularies were discussed. During the discussion, an initial architectural design was collaboratively sketched on a virtual whiteboard to support the discussion. The workshop analysis was carried out by analyzing the notes and assigning the relevant aspects to the questions in the discussion guide.

### Extended Concept

After evaluating the CDSS version 2 prototype, a proposal for an extended concept for future CDSS projects in primary care with the focus on data entry was formulated by MCN in exchange with other authors. This concept considers all the results and describes a design framework that includes UI components, data flows, and system integration.

## Results

### Overview

The conception and development of the UI for a CDSS—in particular, the process of entering data into the CDSS—led to the development of 3 CDSS prototypes (CDSS versions 0, 1, and 2). CDSS version 2 was developed as a high-fidelity prototype connected to AI modules. Evaluation by users and experts revealed several issues regarding user-friendliness, particularly when entering medications and laboratory values. These issues led to recommendations to improve the search function for symptoms and laboratory values, and to integrate medications from the electronic prescription list. Further improvements to data entry could be achieved by integrating the primary care EHR system. EHR system experts considered middleware to be necessary for this, due to the current data exchange format (data exchange family xDT, which is not transferable via the web). In addition, the low level of standardization for important parameters—such as symptoms, which are currently entered as free text—was identified as a challenge. However, current position papers and technical possibilities may simplify this in the future.

### Conception

The results of the conceptualization are presented in 2 tables: Table 2 presents the functional requirements for UI data entry, and Table 3 shows the mapping of the data elements to the UI components and standardization.

Table 3 shows the mapping of the data to be recorded to the UI fields and, if available, the mapping to the standardized international vocabulary of the OMOP database. This is based on the previously defined data model of relevant data entities and vocabularies. The column “mapping to OMOP” represents the mapping to an OMOP standard concept.

Nonstandard vocabularies were mapped in OMOP to SNOMED CT and RxNorm, with the source vocabularies being used in the UI for input. The project aimed to remove free text and to minimize the use of fields not mapped to OMOP.

A few fields were identified as relevant through the requirements analysis, but no direct mapping to OMOP could be established. Symptoms were saved using the HPO vocabulary, which at the time of the project was not supported by OMOP and was not yet available in German. In SATURN, a semiautomatic partial translation was performed for the project using natural language processing [30]. Integration of HPO as vocabulary in OMOP did not take place. HPO and SNOMED CT did not yet have an official mapping at that time—storage therefore took place in the observation table.

**Table 2:** Functional requirements for UI^a^ data entry.

Number	Functional requirement
1	The system must offer the entry of all parameters for diagnoses, procedures, laboratory values, medications, and other clinical entities (in German).
2	The system must contain patient information such as administrative sex and age.
3	The system must generate a unique patient ID for each patient.
4	The system must offer data field-supported input help.
5	The system must ensure the integration of established medical standardized vocabularies used in the data set.
6	The system must allow exclusion diagnoses to be entered in the form (eg, using *ICD*^b^ codes).
7	The system must allow time-based documentation during entry.
8	The system must offer the basic functions of a patient file (create, edit, open, or save).
9	The system must enable the continuous processing of a case and the ability to re-request diagnostic suggestions after a change.
10	The system should offer the transfer of a patient ID from the primary care EHR^c^ system to the SATURN^d^ patient file.
11	The system should offer a parameter query and input support based on previous entries.
12	The system must guarantee the optional input of all data fields.

^a^UI: user interface.

^b^ICD: International Statistical Classification of Diseases and Related Health Problems.

^c^EHR: electronic health record.

^d^SATURN: Smart Physician Portal for Patients With Unclear Disease.

**Table 3:** Mapping of the data basis to the user interface (with German vocabulary).

UI^a^ data category	Mapping to OMOP	Description	UI component	Input format	Additional variables
Patient ID	Person ID	Unique ID of a patient	Auto-generated	Number	—^b^
EHR^c^ system patient ID	Person source value	External unique ID of a patient	Text field	Characters or numbers	—
Gender	SNOMED CT^d^	Administrative gender	Radio button	Selection (female, male, unknown, and other)	—
Date of birth	Date of birth	Patient’s date of birth	Date picker	Datetime (UTC^e^)	—
Postal code	Location ID	Postal code	Text field	Number (5 digits)	—
Diagnosis	SNOMED CT	Diagnosis code, name, and date	Text field with search and date picker	*ICD-10*^f^ code	Date of diagnosis
Symptoms	No mapping to SNOMED CT	Symptoms and date	Text field with search and date picker	HPO^g^ code	Date of symptom onset
Medication	RxNorm	Drug name, and start or end date	Text field with search and date picker	ATC^h^ code	Dose, unit, and days of administration
Vital signs and laboratory values	SNOMED CT	Parameter name, date, and reference range	Text field with search and date picker	LOINC^i^ code	Reference range (only laboratory values) and measurement unit
Procedures	SNOMED CT	Procedure name and date	Text field with search and date picker	OPS^j^ code	Procedure type

^a^UI: user interface.

^b^Not available.

^c^EHR: electronic health record.

^d^SNOMED CT: Systematized Nomenclature of Medicine Clinical Terms.

^e^UTC: Universal Time Coordinated.

^f^ICD-10: International Statistical Classification of Diseases and Related Health Problems, Tenth Revision.

^g^HPO: Human Phenotype Ontology.

^h^ATC: Anatomical Therapeutic Chemical Classification.

^i^LOINC: Logical Observation Identifiers Names and Codes.

^j^OPS: Operation and Procedure Codes (German: “Operationen- und Prozedurenschlüssel”).

### Prototypes

The requirements and the associated vocabularies were implemented step by step. The CDSS version 0 was designed as a low-fidelity prototype in the form of mock-ups [22]. This was followed by the development of the following 2 high-fidelity prototypes: (1) CDSS version 1: JavaScript frameworks nuxt.js 2 (Vuetify), and the UI component framework Vuetify 2 were used with initial sample data (without an active interface to AI modules, as yet, and with integrated vocabularies) [8]. (2) CDSS version 2: React.js (Meta Platforms, Inc) and Mantine were used due to the expiring support of nuxt.js 2. The prototype includes all feasible requirements and has a connection to the AI modules.

**Figure 2:**
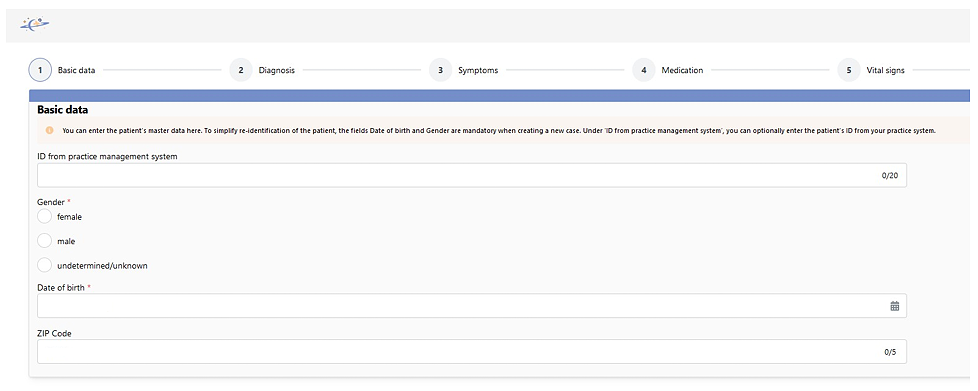
Zoomed-in section of the input template of the CDSS version 2. CDSS: clinical decision support system.

Figure 2 shows the first step of the data entry in the final high-fidelity prototype.

CDSS version 0 mock-ups, as well as screenshots of the 2 high-fidelity prototypes, CDSS versions 1 and 2, can be found in Multimedia Appendix 2. At the request of user feedback on this prototype, a step-by-step navigation was incorporated below (see also Neff et al [8] for a more detailed description of prototype version 1).

**Figure 3:**
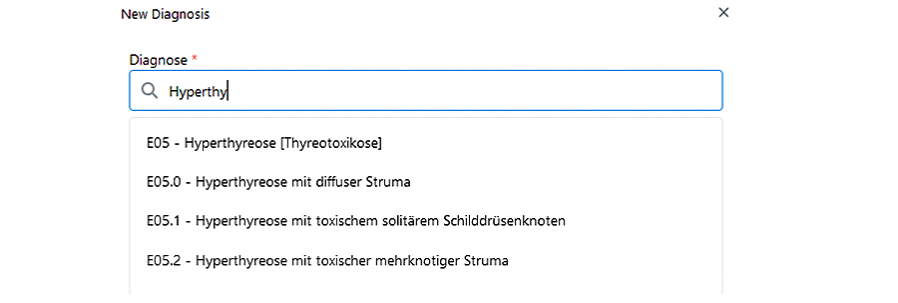
Screenshot of the diagnosis search (CDSS version 2). CDSS: clinical decision support system.

Figure 3 shows an example of how the integration of vocabularies appears using *ICD-10-GM*: users can search for *ICD-10-GM* terms using the code (eg, E05.0) or the disease name (eg, in the German language “Hyperthyreose mit diffuser Struma”).

### System Evaluation Feedback

During the development of CDSS version 1, expert feedback revealed issues, particularly with search field entry options and inconsistencies between system functions and real clinical practice, for example, the handling of “diagnostic certainty.” Feedback also emphasized the importance of automatic saving and clear error messages. Errors were found when entering medications and laboratory values. Some terminology was not displayed in the German language; thus, it is imperative that vocabularies be available in the German language [8]. Detailed information can be found in the publication by Neff et al [8].

**Figure 4:**
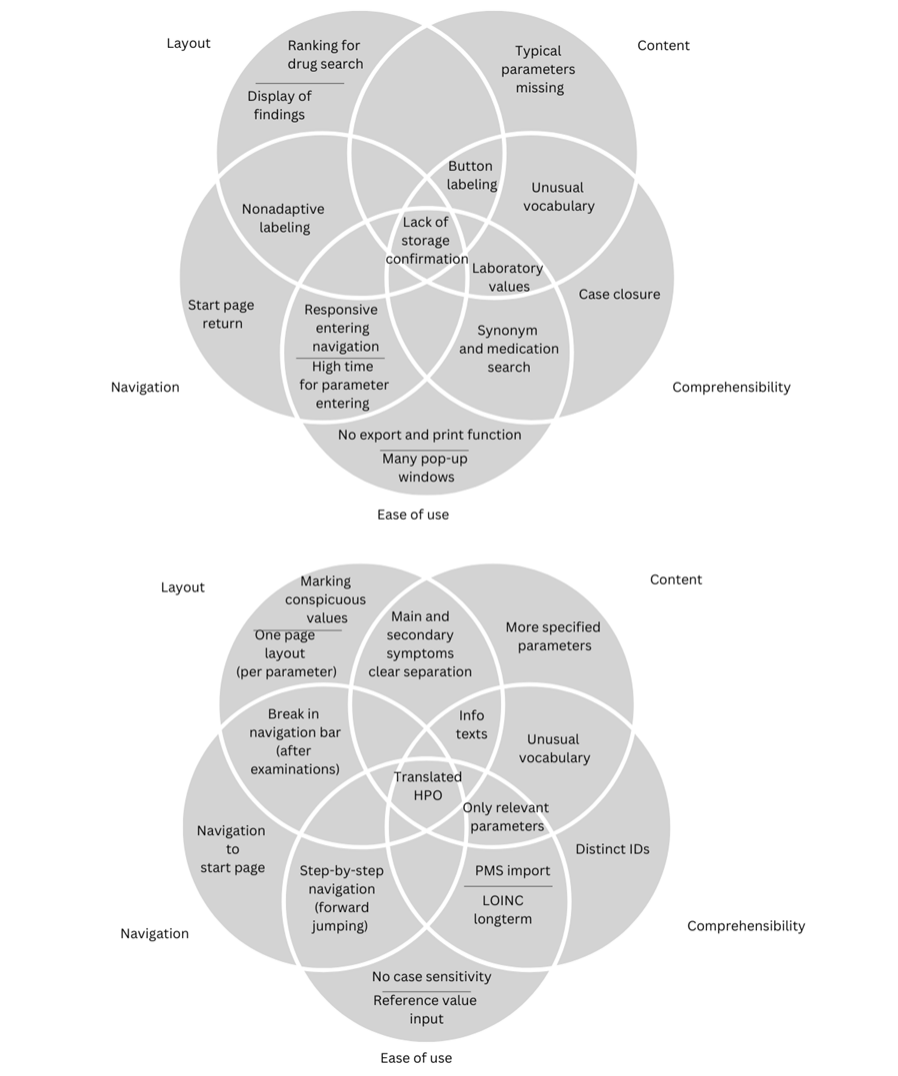
Summarized results of first and second user evaluations (feedback of the first high-fidelity prototype: version 1, and of the final high-fidelity prototype: version 2). CDSS: clinical decision support system; HPO: Human Phenotype Ontology; LOINC: Logical Observation Identifiers Names and Codes; PMS: patient management system.

In addition to expert feedback from the inspections, further issues were derived from the user evaluations. Feedback on data entry was collated into categories (layout, content, navigation, comprehensibility, and ease of use). The results of the CDSS version 1 usability test (Figure 4) indicated several usability issues. Participants had difficulties entering unlisted symptoms, medications, and tests, and retrieving laboratory values using common abbreviations. Medication data was the biggest problem area. The wide range of laboratory units also caused confusion; thus, specifications for values such as the heart rate were proposed. Some important GPs’ diagnostic criteria were absent, with participants proposing their inclusion within the system or the creation of additional free entry fields. The additional fields were proposed to encompass physical examination findings, symptom duration, diagnoses, and family history. GPs also proposed issues for the CDSS version 2 (Figure 4), including automated display of reference range and integration of an updated medication database. GPs were unfamiliar with LOINC (Logical Observation Identifiers Names and Codes) terms, and so better input support was recommended. Participants also recommended focusing on frequency, time, dosage, and extending the search function. This would allow GPs to enter both generic and brand names, as data entry for laboratory values, medications, and diagnoses was considered time-consuming. The information text to each input step was unclear and not sufficiently relevant, and the distinction between some parameter names was not apparent. The GPs also noted several limitations; these included the inability to enter lowercase letters for laboratory reference values and the failure of the symptom search function to recognize certain synonyms.

### Workshop With EHR System Experts

The workshop was attended by a total of 12 participants (7 women and 5 men): (1) four project representatives (experts with backgrounds in medicine, medical informatics, medical technology, or public health) from the SATURN project; (2) five representatives from three different companies in the field of EHR systems for primary care (experts for EHR systems); (3) the workshop confirmed that currently, the most widely used data transmission standard (syntactically) in primary care in Germany is still the xDT data standard family [49]. These include, in particular: Behandlungsdaten-Transfer (further development completed in 2019) for the transfer of treatment data [50], and Labordaten-Transfer for laboratory values [51].

Both standards are defined by the KBV (Kassenärztliche Bundesvereinigung; umbrella organization of the Associations of Statutory Health Insurance Physicians in Germany) [52]. The data transfer (structural) is currently realized via xDT interfaces and is not transferable via the web. A 2022 position paper ([53]) indicated movement toward FHIR (Fast Healthcare Interoperability Resources from HL7 [Health Level Seven]) and thus web transfer. However, the workshop participants have indicated that not all the necessary FHIR profiles have yet been specified. The JSON format was considered a favorable option for the future, with or without a combination with FHIR. Initial data, such as the MIO “Medizinische Informationsobjekte,” representing the new KBV data exchange standard [54], are already available in JSON. Table 4 summarizes the terminology and data structure (semantics) for the participants’ description of the status quo for the specific CDSS for the primary care use case. All primary care EHR systems are required to provide an electronic physician letter, as part of the telematics infrastructure (TI) in Germany [55].

Participants concluded based on the status quo that middleware is currently indispensable in Germany for integrating EHR systems with CDSS in primary care due to limited data structures and limited data transfer options. Middleware is required to map data to standard medical terminologies used in the CDSS. Ideally, an interface for diagnoses (billing diagnoses in *ICD-10-GM*), laboratory values, and the electronic physician letter should be available via the EHR system. The CDSS should ensure the addressability of the interface and the import of medications from the electronic medication plan. The electronic physician’s letter can be used to extract symptoms and examinations. If the electronic physician letter cannot be sent through the EHR system, a new interface is needed. In the future, other medical devices and wearables will connect to the CDSS via middleware. The workshop participants also provided the following feedback on the potential for future developments: data processing with AI is already partially integrated into the EHR systems in primary care. TI measures have also been implemented and are mandatory for manufacturers in Germany.

These include, besides the physician’s electronic letters, electronic medication plans and electronic prescriptions, which are set to increase. Connecting the EHR system to other systems, such as the CDSS, is considered feasible and can be implemented depending on the availability and format of data, using REST (Representational State Transfer), a software architecture paradigm.

**Table 4:** Semantic description of status quo: terminologies and structured data in primary care EHR^a^.

Data category	Status quo (data structure)
Basic patient data	Patient data, such as date of birth and postcode, are considered primitive data types and, therefore, are easy to structure and transfer via an interface.
Diagnosis	Diagnoses are structured as billing and permanent diagnoses, with some stored as “suspected diagnoses” and localizations. Transferring the *ICD-10-GM*^b^ codes is, according to the current state of the art, challenging.
Symptoms	Symptoms are typically documented as free text, using, for example, nonstandardized categories in chronological index cards.
Medication	Medications are ideally coded using national medication plans (ATC^c^ codes or PZN^d^); EHR systems must implement the standardized national medication plan, which has been mandatory since 2016 [56].
Laboratory and vital signs	Laboratory values comply with the LDT^e^ 3 standard, although many laboratories continue to use unstructured data that are not LOINC^f^ compliant [52,57]. The KBV^g^ is developing an MIO^h^ for laboratory values [54]. Vital signs can be recorded as either discrete or continuous measurements for billing purposes.
Procedures	Procedure codes (eg, OPS^i^) are structured in hospital outpatient departments but are rarely used in primary care EHR systems; outpatient procedures are often documented as service code justifications for billing purposes.

^a^EHR: electronic health record.

^b^*ICD-10-GM*: *International Statistical Classification of Diseases and Related Health Problems, Tenth Revision, German Modification*.

^c^ATC: Anatomical Therapeutic Chemical Classification.

^d^PZN: Pharmazentralnummer.

^e^LDT: Labordaten-Transfer.

^f^LOINC: Logical Observation Identifiers Names and Codes.

^g^KBV: Kassenärztliche Bundesvereinigung.

^h^MIO: Medizinische Informationsobjekte.

^i^OPS: Operation and Procedure Codes (German: “Operationen- und Prozedurenschlüssel”).

### Extended Concept

#### Overview

The concept is described in the form of a design framework with UI components, data flows, and system integration.

#### UI Components

**Figure 5:**
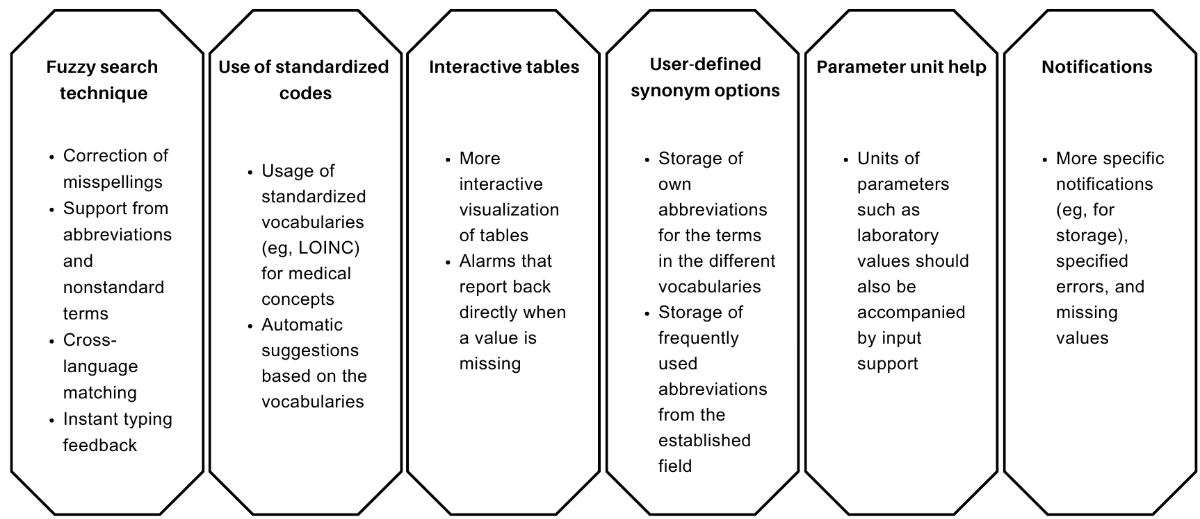
UI component optimizations. LOINC: Logical Observation Identifiers Names and Codes; UI: user interface.

The required ontologies will continue to be entered as standardized vocabulary, but will be supported by various factors. These include error-tolerant searching that ignores misspellings and similar spellings, and the use of common abbreviations [58-60]. These approaches are visualized in Figure 5.

#### Data Flows

The input of patient data should be possible both via the UI and via an interface to the primary care EHR system or TI applications. Supporting middleware can help with this, especially for laboratory values and medications. The data flow then goes from the UI to the backend for storage, which is connected to the stored database. A data flow from the database via the backend to the UI occurs only when displaying and correcting previously saved cases or when retrieving the vocabularies for input support.

#### System Integration

System integration is provided by a REST interface from the UI to the backend and REST interfaces to the AI modules.

## Discussion

### Discussion of Methods

The objective of this study was to investigate how the UI of a CDSS designed for primary care can effectively support GPs in the user-friendly input of standardized vocabulary codes from medical terminologies. It focuses on the CDSS UI, as well as the usability and clarity of medical terminology input.

Methods from software development and UCD were used as part of the iterative process. The UCD design process has been proven to have positive effects in several studies [61-64]. Clinical decision support is generally viewed favorably, providing higher benefits when it improves performance [63]. The current study addresses the integration of the CDSS into the physicians’ workflow, an aspect shown in the literature to be essential for CDSS acceptance and usage [65]. To simplify the input of medical terms into the UI for GPs, the UI must be designed to be supportive and user-friendly [20]. One key aspect, therefore, is the reduction of cognitive load. This is described by Miller et al [65] as an important principle for CDSS design: focusing on the input of standardized medical terminology codes can help to reduce the cognitive load on physicians.

The research conducted in this project and other studies has mainly focused on UCD, requirements analysis, and evaluation of CDSS [8,21,63,66]. The formulation of functional requirements and task model methods has proven effective [39,67]. Building on these results, the current study mapped relevant data elements to the UI and collaborated with primary care EHR system experts to develop an extended concept. Zerlik et al [68] took a similar approach in their initial steps, following a UCD process that included requirements definition and iterative development across 3 iterations. We complemented usability expert testing and workshops with feedback from EHR system experts. This approach enabled us to identify potential interfaces to other systems, consistent with recommendations that include different perspectives in the design process [64].

### Discussion of the Results

This study identifies requirements and options for entry support in the UI of a primary care CDSS and provides a conceptual framework to support future CDSS implementations. The feedback from the usability test indicates that optimized input support in the language of GPs will be necessary in the future, as will additional mapping work. It is imperative to avoid unfamiliar terms and minimize input restrictions. Ideally, the system would integrate directly with the primary care EHR system in use. The workshop with primary care EHR system experts resulted in initial solution approaches for system interfaces. However, it also highlighted challenges related to limited interoperability, including a simple nonweb-transferable data exchange format and a lack of standardization for important parameters. We propose various optimizations for the UI in our concept for a future CDSS UI. There are several options to better support standardized data entry. Standardizing data entry and using automated tools can significantly improve data quality by minimizing the need for queries and simplifying data collection. Automated features, such as auto-complete, can improve accuracy by relying on predictive modeling. Integration with generative AI can further improve data accuracy and usability. Nevertheless, further research is needed to optimize the role of standardized data in clinical decision-making and in the research context [69]. AI (eg, machine learning) and natural language processing methods already exist for data integration and mapping, data extraction, and clinical coding.

Models for clinical data extraction, phenotyping, and LOINC mapping also exist [70-74]. A mandatory opt out system for the electronic patient records (ePA) planned for 2025 in Germany, focusing on secure storage and pseudonymized data usage, may facilitate implementing an interface to the ePA data [75,76]. The Unified Medical Language System metathesaurus is another option, already being partially in use, that supports terminology mapping and standardization and enables better interoperability and information retrieval [77]. When searching the medical literature, it is often easier to use the language you are most familiar with. Using one’s native language can facilitate the identification of certain concepts that are difficult to translate into English [60]. There is still a need for improvement in this area, particularly regarding the German-language description of symptoms. Further development of standards and translations, as well as data transfer formats such as FHIR for connecting primary care EHR to CDSS, may simplify integration in the future. When implementing in practice, it is important to incorporate the system into the daily workflow and connect it to the practice’s own EHR system.

### Limitations

This study focused on integrating medical terminologies into the UI, representing 1 task within the task model. The methodological framework was designed to address this objective. However, the steps are transferable to other tasks within the task model. This requires selecting the requirements and involving additional experts (such as EHR system experts) relevant to the task. Further studies in real-world clinical settings, using the implemented system over an extended period, are necessary to develop the CDSS. For the specific task of entering medical terminology into the CDSS, fuzzy search can provide excellent support; however, there are also challenges in the form of high computational effort, false positive results, and balanced precision in different languages [58]. For the integration into practice, the current infrastructure and networking in Germany make it difficult to interface with the primary care EHR system. The slow uptake of ePA in Germany, with only ≈1% penetration by 2023 compared to a target of 80% by 2025, is hampering automation. Barriers to adoption include legal ambiguities, conflicts between stakeholders, and insufficient communication [75,76].

Thanks to their modular structure, the practices will not require significant resources. However, the task here is to ensure secure implementation, including hosting of the AI modules and secure data transfer.

### Conclusion

In conclusion, the process of UCD has been shown to be effective in the development of the UI of CDSS, particularly regarding the integration into physicians’ workflow and creation of a user-friendly interface. Iterative development with experts identified potential interfaces for other systems. The results indicated a need for optimized entry support in the physician’s language and revealed challenges concerning interoperability. Suggestions for improvements include standard data entry, automated tools, and AI to enhance data quality and usability. However, the current infrastructure poses significant challenges for interfacing with primary care EHR systems in Germany. Further studies are required to address these issues.

## Appendix

Multimedia Appendix 1 Discussion guide of EHR expert workshop. EHR: electronic health record.

Multimedia Appendix 2 Screenshots of the CDSS user interface (prototypes: CDSS versions 0, 1, and 2). CDSS: clinical decision support systems.

## Abbreviations

AI: artificial intelligence
CDM: Common Data Model
CDSS: clinical decision support systems
DIC: Data Integration Centre
EHR: electronic health record
ePA: electronic patient record
FHIR: Fast Healthcare Interoperability Resources
GP: general practitioner
HL7: Health Level Seven
HPO: Human Phenotype Ontology
ICD-10-GM: International Statistical Classification of Diseases and Related Health Problems, Tenth Revision, German Modification
ISO: International Organization for Standardization
KBV: Kassenärztliche Bundesvereinigung
LOINC: Logical Observation Identifiers Names and Codes
OPS: Operation and Procedure Codes (German: “Operationen- und Prozedurenschlüssel”)
REST: Representational State Transfer
SATURN: Smart Physician Portal for Patients With Unclear Disease
SNOMED CT: Systematized Nomenclature of Medicine Clinical Terms
TI: telematics infrastructure
UCD: user-centered development process
UI: user interface
