# Supplementary material for: Experiences With Integrating Medical Terminologies Into User Interfaces for a Decision Support System for Primary Care: Conceptual and Development Study
Source: JMIR Med Inform. 2026 Feb 20;14:e74934. doi: 10.2196/74934 (PMC12966822; doi:10.2196/74934)
Supplement: Multimedia Appendix 1 [file medinform_v14i1e74934_app1.docx]

| **Question** | **Subquestion(s)** | **Category** |
| --- | --- | --- |
| Introductory question:  How would you imagine a simple interface between a  a primary care EHR system and a decision support system? | - Imagine a variant that is as simple as possible - what is your first thought on this topic? - What ideas and experiences do you have? | First ideas and experiences |
| Which data standard do you suggest for the EHR system to CDSS interface? | - What are the data standards you use for interfaces? - Which data standard would you suggest for use in this specific application? | Syntactic level |
| Are the types of data we have mentioned available for exchange in a structured way in the EHR system? | - Is the data mentioned based on vocabularies or can it be mapped with them? | Semantic level |
| How should data be transferred? | - From a technical point of view, how can data be exchanged with a specific interface? | Structural level |
| What might a specific data query look like? | - What are the options for providing and receiving data from the primary care EHR system? | Process interoperability |
| Outlook: What will change in the future? | - How will data standards change? - How realistic is the future connection of e.g. diagnostic support systems? | Implementation and outlook |
