# Supplementary material for: Experiences With Integrating Medical Terminologies Into User Interfaces for a Decision Support System for Primary Care: Conceptual and Development Study
Source: JMIR Med Inform. 2026 Feb 20;14:e74934. doi: 10.2196/74934 (PMC12966822; doi:10.2196/74934)

Prototypes of the CDSS UI for primary care - Task: Data entry

CDSS v0: Low-fidelity prototype

**Mock-up S1.** Basic data/patient record entry.


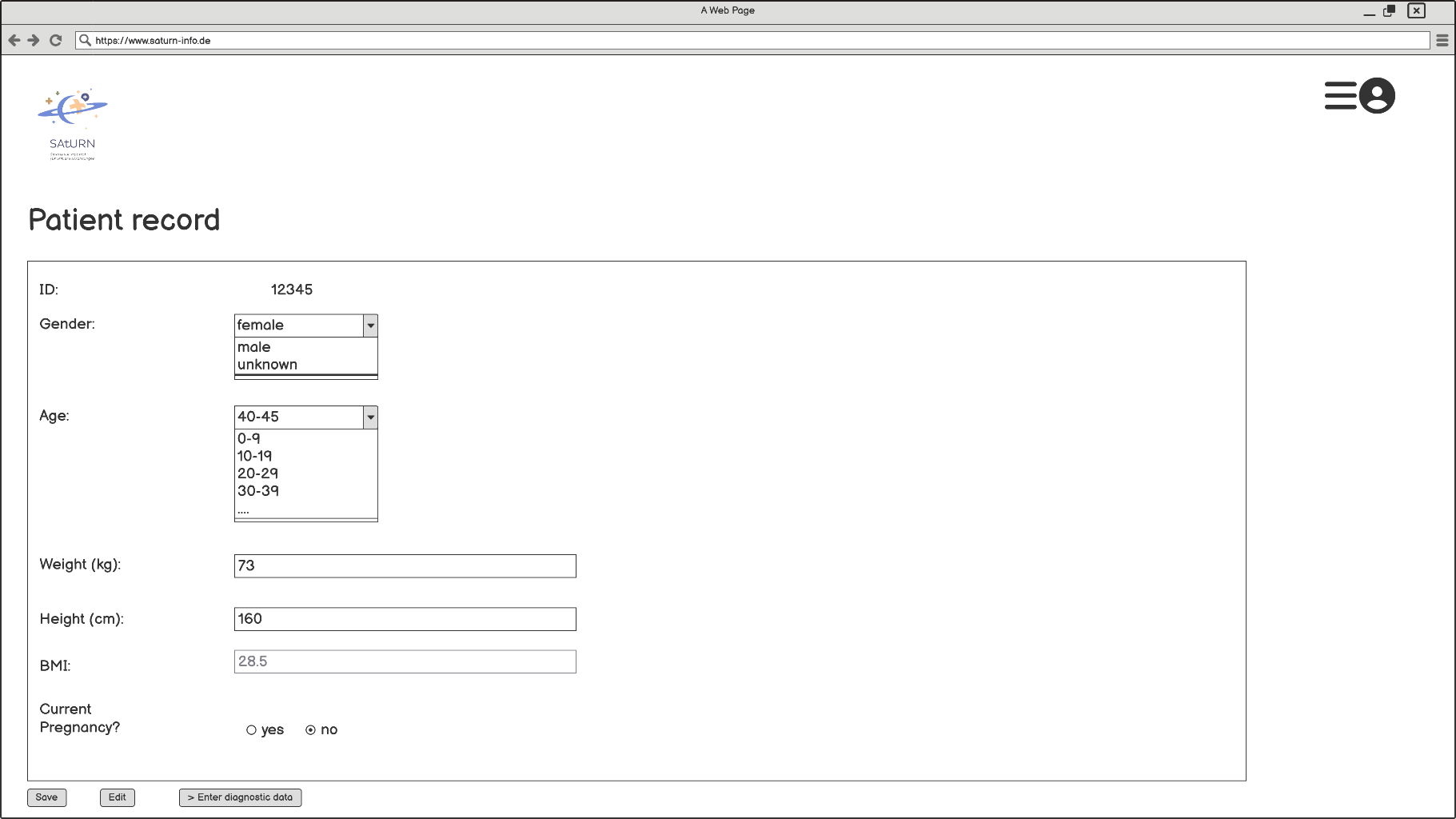


**Mock-up S2.** Diagnostic data entry.


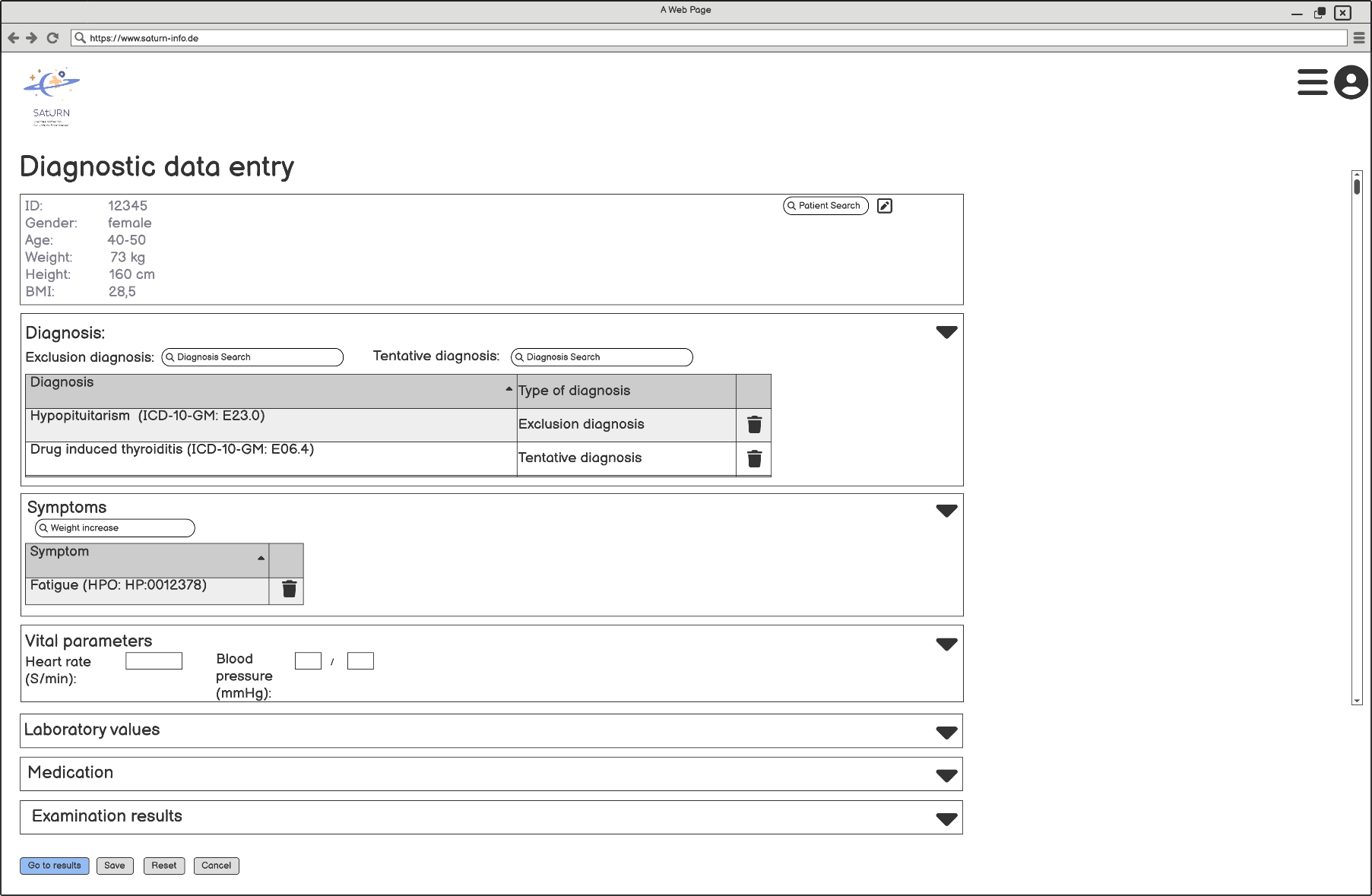


CDSS v1: First high-fidelity prototype

**Figure S1.** Basic data entry.


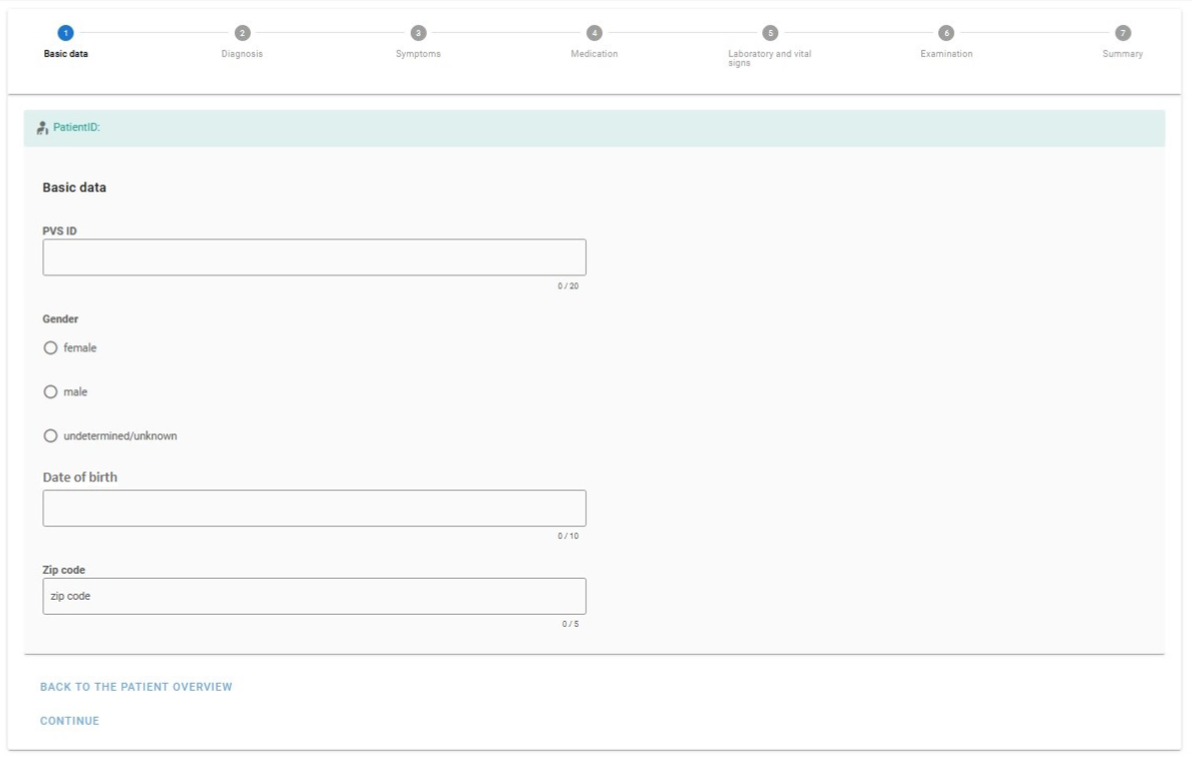


**Figure S2.** Diagnostic data entry—step: laboratory and vital sign values.


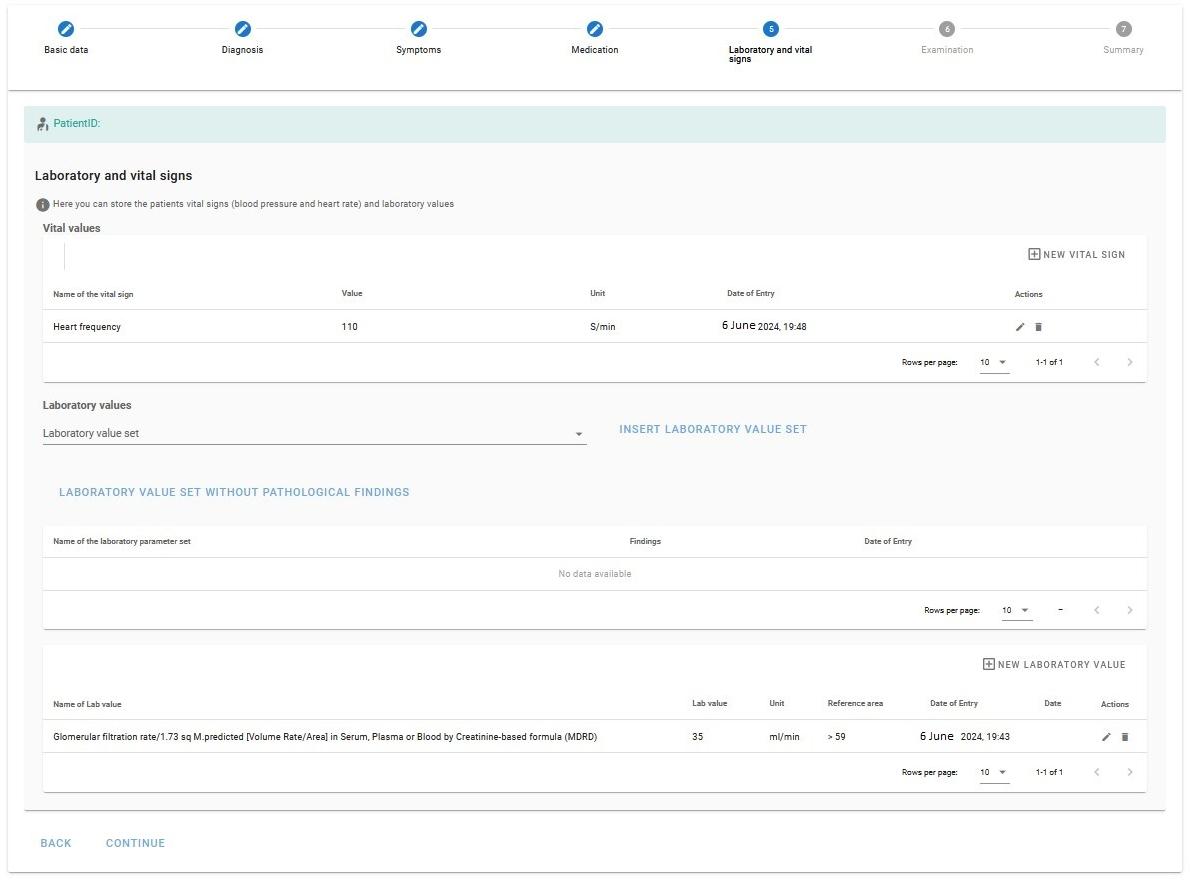


CDSS v2: Final high-fidelity prototype

**Figure S3.** Basic data entry.


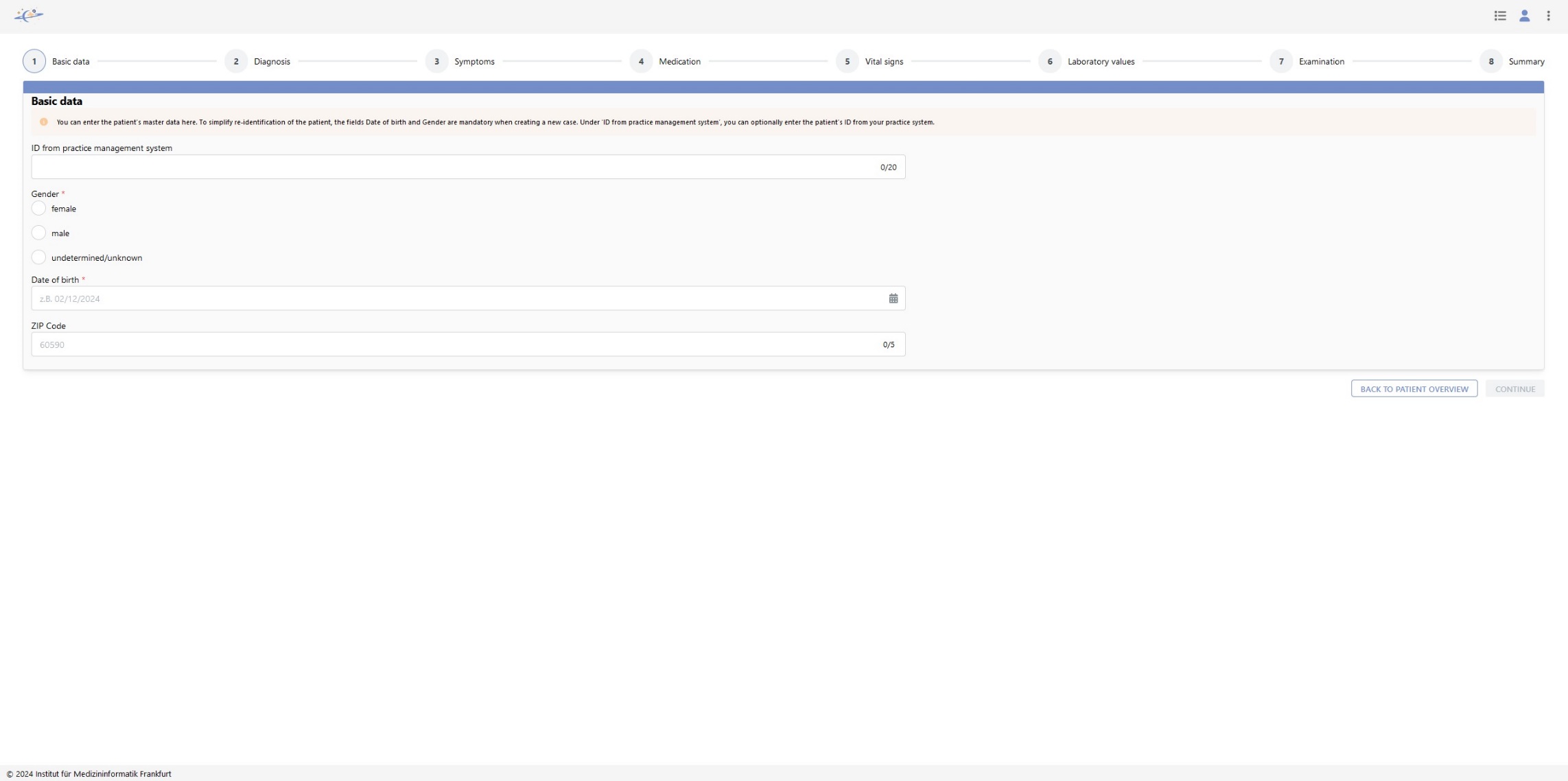


**Figure S4.** Diagnostic data entry—step: laboratory and vital sign values.


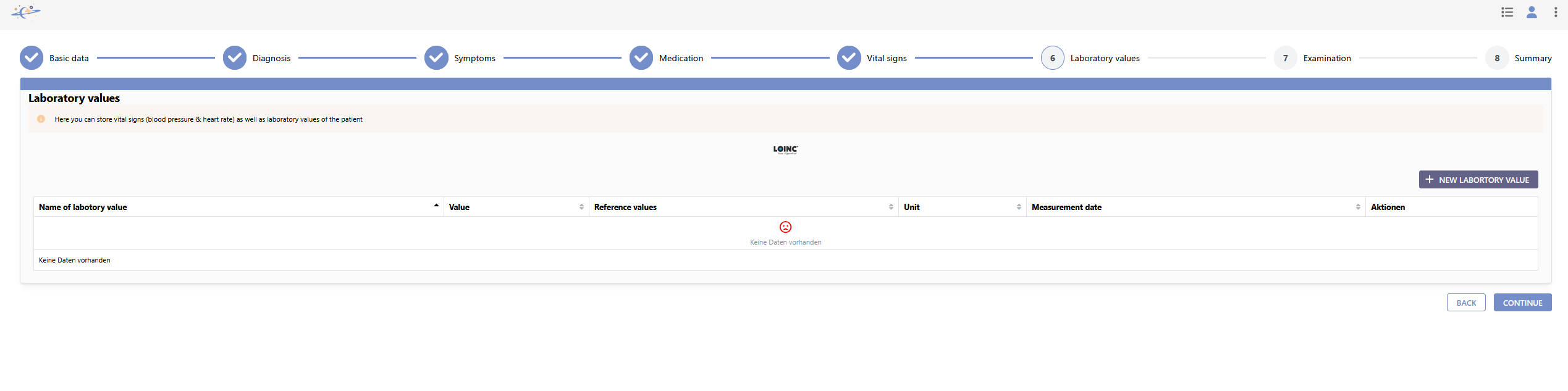

Supplement: Multimedia Appendix 2 [file medinform_v14i1e74934_app2.docx]
